# Culprit-only versus staged complete revascularization for patients with ST-segment elevation myocardial infarction and Multivessel disease: a retrospective cohort study

**DOI:** 10.1186/s12872-016-0365-5

**Published:** 2016-10-06

**Authors:** Tongtong Yu, Yuanyuan Dong, Jiahe Zhu, Chunyang Tian, Zhijun Sun, Zhaoqing Sun

**Affiliations:** Department of Cardiology, Shengjing Hospital of China Medical University, Shenyang, Liaoning People’s Republic of China

**Keywords:** Multivessel disease, Revascularization, ST-segment elevation myocardial infarction

## Abstract

**Background:**

Multivessel disease (MVD) is common in patients with ST-segment elevation myocardial infarction (STEMI), but optimal treatment management remains undetermined.

**Methods:**

In this retrospective cohort study, 602 consecutive STEMI patients with MVD were enrolled between January 1, 2010 and October 1, 2014. Three hundred and eighty-two patients underwent culprit-only revascularization and 220 underwent staged complete revascularization. Primary end points were a composite of cardiac mortality or nonfatal reinfarction.

**Results:**

The mean duration of follow-up was 35 months (12–71 months). Following multivariate analysis, staged complete revascularization was associated with a lower rate of the composite of cardiac mortality or nonfatal reinfarction [HR: 0.430, 95 % CI: 0.197–0.940, *P* = 0.034] and unplanned repeat revascularization [HR: 0.343, 95 % CI: 0.166–0.708, *P* = 0.004] compared with culprit-only revascularization.

**Conclusions:**

Compared with culprit-only revascularization, staged complete revascularization significantly reduced the rate of the composite of cardiac mortality or nonfatal reinfarction, and the need for unplanned repeat revascularization.

## Background

Primary percutaneous coronary intervention (P-PCI) of the culprit artery is widely used in patients with ST-segment elevation myocardial infarction (STEMI). Approximately 50 % of STEMI patients have multivessel disease (MVD) [1, 2]. Non-culprit lesions are not just “bystanders”, as a pathophysiological inflammation process in acute myocardial infarction could cause plaque instability [3, 4]. Previous research has also shown that STEMI patients with MVD have higher mortality rates and a greater incidence of non-fatal reinfarction than those without MVD [1, 2]. However, the optimal management of STEMI patients with MVD remains undetermined [5–7]. Although a number of randomized controlled trials (RCTs) [8–11], including the PRAMI [9], CVLPRIT [10] and DANAMI-3—PRIMULTI [11] trials, have indicated the clear benefits of complete PCI, other RCTs [12–14], including the PRAGUE-13 trial [12], found no difference between complete and culprit-only revascularization in STEMI patients with MVD. Furthermore, observational studies [15–21] and meta-analyses [22–24] also demonstrated conflicting results.

The present study aimed to determine the benefits and safety of staged complete revascularization in STEMI patients with MVD undergoing P-PCI.

## Methods

### Study design and setting

This was a retrospective cohort study, and included consecutive STEMI patients who were hospitalized and underwent PCI at Shengjing Hospital of China Medical University (Shenyang, China) between January 1, 2010 and October 31, 2014. Six hundred and two consecutive cases were selected in this large-scale hospital in Northeast China. Firstly, the investigators identified all consecutive PCI patients from PACS (Picture Archiving and Communication Systems) of the interventional imaging data and assigned each case a unique study ID. The investigators then abstracted comprehensive clinical data and procedural data using electronic medical records. Abstracted elements included patient demographic characteristics, past cardiac and noncardiac history, patient clinical characteristics on hospital admission, laboratory measurements, procedure-related complications and use of cardiac medications during the index hospitalization and at discharge. Killip classification was introduced [5]. All venous blood samples were obtained on admission and tested using autoanalyzers in the core laboratory of Shengjing Hospital and standard techniques. Left ventricular ejection fraction (LVEF) was determined by echocardiography during hospitalization. Procedural data from surgical records in PCI cases were completed by operators. Angiographic variables were estimated visually or by a quantitative computer analysis system. Thrombolysis In Myocardial Infarction (TIMI) flow grade was determined as defined previously [25]. Clinical follow-up was assessed in October 2015 by hospital visits or phone interviews with the patient’s general practitioner/cardiologist, the patient or his/her family. All events were obtained from the patients’ medical records. If these data were unavailable, statuses were ascertained by a telephone call to the patient’s referring hospital physician. All events were adjudicated and classified by two cardiologists.

### Participants and procedures

We identified 1056 STEMI patients treated with P-PCI. Patients who were eligible for P-PCI met the following criteria: (1) chest pain present less than 12 h from onset of pain to time of catheterization, (2) significant ST-segment elevation (at least 0.1 mV in two or more standard leads or at least 0.2 mV in two or more contiguous precordial leads) or a new left bundle branch block. After confirmation of STEMI, P-PCI was immediately undertaken according to current guideline recommendations and operators’ routine practice. Operators decided on the use of aspiration thrombectomy, heparin, or glycoprotein IIb/IIIa inhibitor. The culprit artery was determined using ECG, echocardiography and angiographic findings by each operator. For inclusion in the present study, patients had to have MVD, which was defined as the presence of angiographic diameter stenosis of 50 % or greater in at least one non-culprit major epicardial coronary artery or its major branches (with diameter ≥2 mm). Exclusion criteria included (1) single vessel disease, (2) cardiogenic shock, (3) any type of stent thrombosis, (4) previous coronary artery bypass grafting (CABG), (5) unsuitable for treatment with P-PCI, (6) chronic total occlusion as the only significant non-culprit lesion, (7) non-culprit lesion in coronary artery branches 2 mm or smaller in diameter. The study population was subdivided into (1) the culprit-only revascularization group (CR group), in which only the culprit lesion received PCI during the index catheterization or hospitalization; (2) the staged complete revascularization group (SR group), in which, after culprit lesion PCI, a planned additional non-culprit lesion PCI was performed during the index hospitalization, or within 1 month after discharge, regardless of symptoms or evidence of ischemia. Periprocedural and postprocedural anti-platelet treatments and other cardiovascular medications were administered in accordance with current guidelines [5, 7].

### Clinical end points

The primary end point was a composite of cardiac mortality or nonfatal reinfarction. Secondary end points were all-cause mortality, cardiac mortality, nonfatal reinfarction and unplanned repeat revascularization, including any unplanned repeat PCI or surgical bypass of target or non-target vessels. The safety end points were periprocedure-related complications, including BARC 3 or 5 bleeding, contrast-induced nephropathy, stroke, and acute or subacute stent thrombosis during the index hospitalization. Stroke was defined as an acute event of non-hemorrhagic cerebrovascular origin causing focal or global neurologic dysfunction lasting >24 h, which was confirmed by both clinical and radiographic criteria. Contrast-induced nephropathy was defined as an increase in serum creatinine concentration ≥0.5 mg/dl (44.2 mmol/l) or ≥25 % above baseline 72 h after exposure to the contrast medium. All other end points were defined by standardized definitions [26, 27]. This study complies with the Declaration of Helsinki, and Shengjing Hospital of China Medical University Research Ethics Committee approved the research protocol. Written informed consent was formally obtained from all participants.

### Statistical analysis

Quantitative variables with normal distribution were represented as mean ± standard deviation (SD) and compared with the independent samples t-test. Quantitative variables without normal distribution were represented as median [interquartile range, IQR] and compared with the Mann-Whitney U-test. Normal distribution was assessed by the one-sample Kolmogorov-Smirnov Test. Categorical variables were represented as counts and proportions (%) and compared using the chi-square test. Event-free survival was estimated in the two groups from Kaplan–Meier curves and compared using the Log-Rank Test. Cox proportional-hazards regression modeling was used to analyze the effects of variables on event-free survival. Variables in Table 1 with *P* ≤ 0.1 at the univariate analysis were “entered” into the model (Table 3). These variables included age, gender, current smoker, and previous MI. Results were reported as hazard ratios (HRs) with associated 95 % confidence intervals (CIs). All tests were two-sided, and the statistical significance was defined as *P* < 0.05. All statistical analyses were performed using SPSS version 19 (SPSS Inc., Chicago, Illinois, USA).

**Table 1:** Demographics and baseline clinical characteristics, means ± SD, or *N* (%)

	CR, *n* = 382	SR, *n* = 220	*P*
Age, yrs	64.6 ± 12.0	62.7 ± 11.5	0.052
Male	257 (67.3)	164(74.5)	0.061
Medical history			
Diabetes	101 (26.4)	70 (31.8)	0.159
Hypertension	194 (50.8)	120 (54.5)	0.374
Hypercholesterolemia	100 (26.2)	56 (25.5)	0.845
Current smoker	194 (50.8)	128 (58.2)	0.080
Previous PCI	14 (3.7)	10 (4.5)	0.595
Previous MI	13 (3.4)	14 (6.4)	0.091
Killip class II/III on admission	27 (7.1)	13 (5.9)	0.582
Systolic blood pressure on admission, mmHg	128.2 ± 22.0	129.9 ± 24.0	0.392
Heart rate on admission, bpm	77.3 ± 16.8	77.8 ± 14.5	0.703
LVEF, %	54.0 ± 9.1	53.6 ± 9.1	0.662
Symptom to balloon time, h	6 (4,9)	6 (3,9)	0.851
Anterior MI	165 (43.2)	103 (46.8)	0.389
Three-vessel disease	160 (41.9)	106 (48.2)	0.134
Intra-aortic Balloon Pump	31 (8.1)	17 (7.7)	0.866

*MI* myocardial infarction, *bpm* beats per minute, *h* hour

## Results

### Participants

**Fig. 1:**
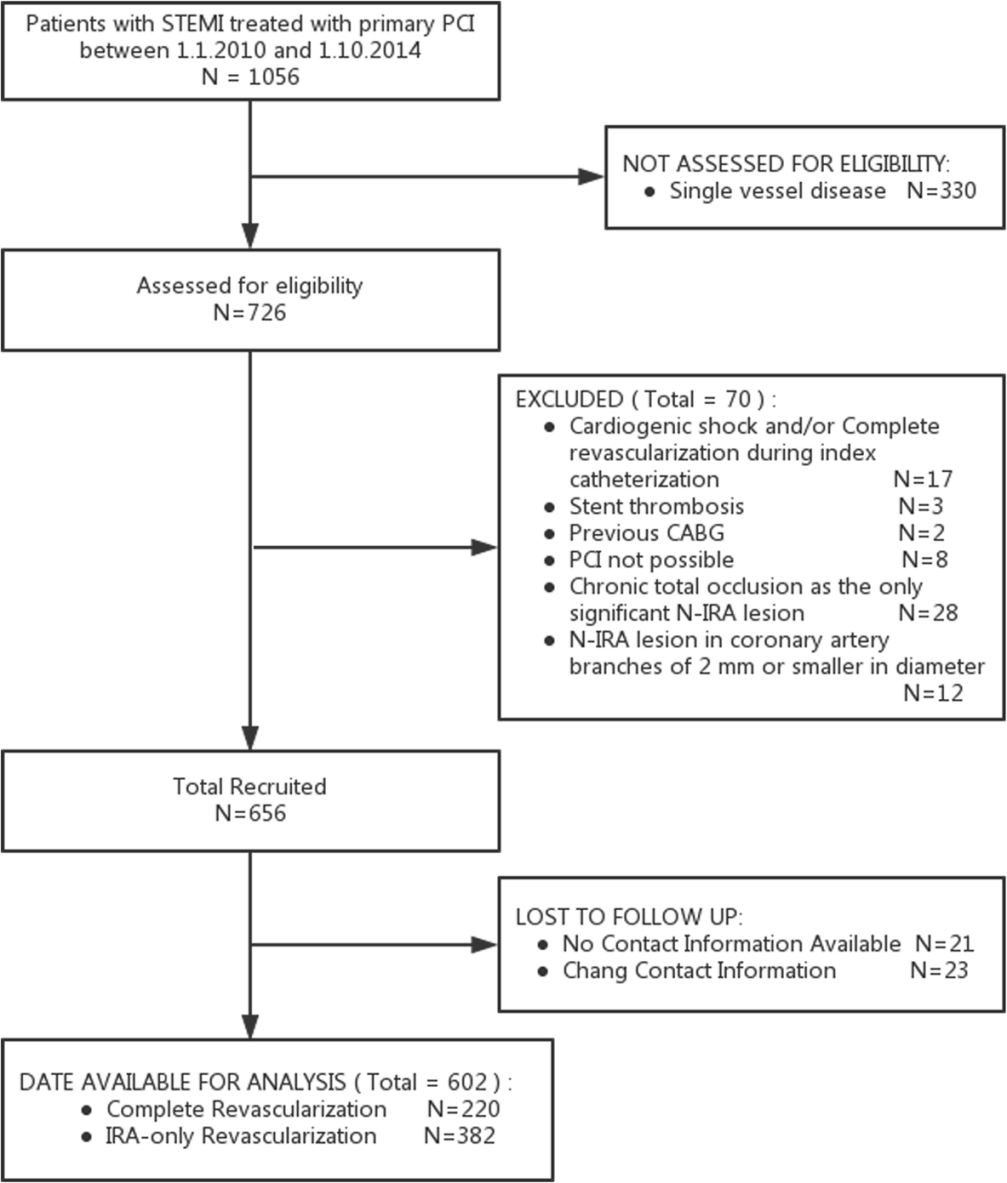
Flow diagram of participant selection. 330 with single vessel disease, 70 with other exclusion criteria, and 44 without follow-up were excluded. The final study cohort consisted of 602 patients, of whom 382 received culprit-only revascularization and 220 received staged complete revascularization. STEMI, ST-segment elevation myocardial infarction; PCI, percutaneous coronary intervention; CABG, coronary artery bypass grafting; N-IRA, non-Infarct-Related Artery

Between January 1, 2010 and October 1, 2014, a total of 1,056 patients were treated with P-PCI for STEMI in our center. Figure 1 represents the flowchart for patient selection. The final study cohort consisted of 602 patients, of whom 382 (63.5 %) received culprit-only revascularization and 220 (36.5 %) received staged complete revascularization. For the SR group, the timing of non-culprit lesion PCI was during the index hospitalization using a staged procedure (*n* = 208) and after index hospitalization but within 1 month (*n* = 12).

### Basic characteristics

Clinical characteristics in the two groups were generally similar and are shown in Table 1. Periprocedural details and discharge medication are shown in Table 2. Patients in the SR group had more stents and longer total stent length. Discharge medication was similar between the two groups (Table 2).

**Table 2:** Periprocedural details and discharge medication, median (IQR), or *N* (%)

	CR, *n* = 382	SR, *n* = 220	*P*
Percutaneous coronary intervention			
TIMI flow grade 0/1 on arrival	288 (75.4)	165 (75.0)	0.914
TIMI flow grade 3 post-PCI	375 (98.2)	218 (99.1)	0.369
Number of stents	1 (1,2)	3 (2,4)	<0.001
Stent type			0.211
No stenting	9 (2.4)	1 (0.5)	
Bare metal	2 (0.5)	1 (0.5)	
Drug-eluting	371 (97.1)	218 (99.1)	
Total stent length for all lesions treated, mm	36 (24,57)	79 (54,109)	<0.001
Lesion site in culprit vessel			0.700
Left anterior descending artery	169 (44.2)	95 (43.2)	
Left circumflex artery	48 (12.6)	33 (15.0)	
Right coronary artery	165 (43.2)	92 (41.8)	
Thrombus aspiration catheter used	55 (14.4)	27 (12.3)	0.464
Use of glycoprotein IIb/IIIa inhibitor	142 (37.2)	127 (42.3)	0.217
Medical treatment at discharge			
Aspirin	376 (98.4)	217 (98.6)	0.840
Clopidogrel	373 (97.6)	213 (96.8)	0.544
Ticagrelor	5 (1.3)	5 (2.3)	0.373
Statin	358 (93.7)	203 (92.3)	0.498
Beta-blockers	224 (58.6)	115 (52.1)	0.121
Angiotensin-converting enzyme inhibitors/Angiotensin receptor blockers	224 (58.6)	133 (60.5)	0.662
Calcium-channel blocker	24 (6.3)	9 (4.1)	0.255
Nitrate	39 (10.2)	16 (7.3)	0.228
Nicorandil	20 (5.2)	6 (2.7)	0.145

### Clinical Outcome

**Fig. 2:**
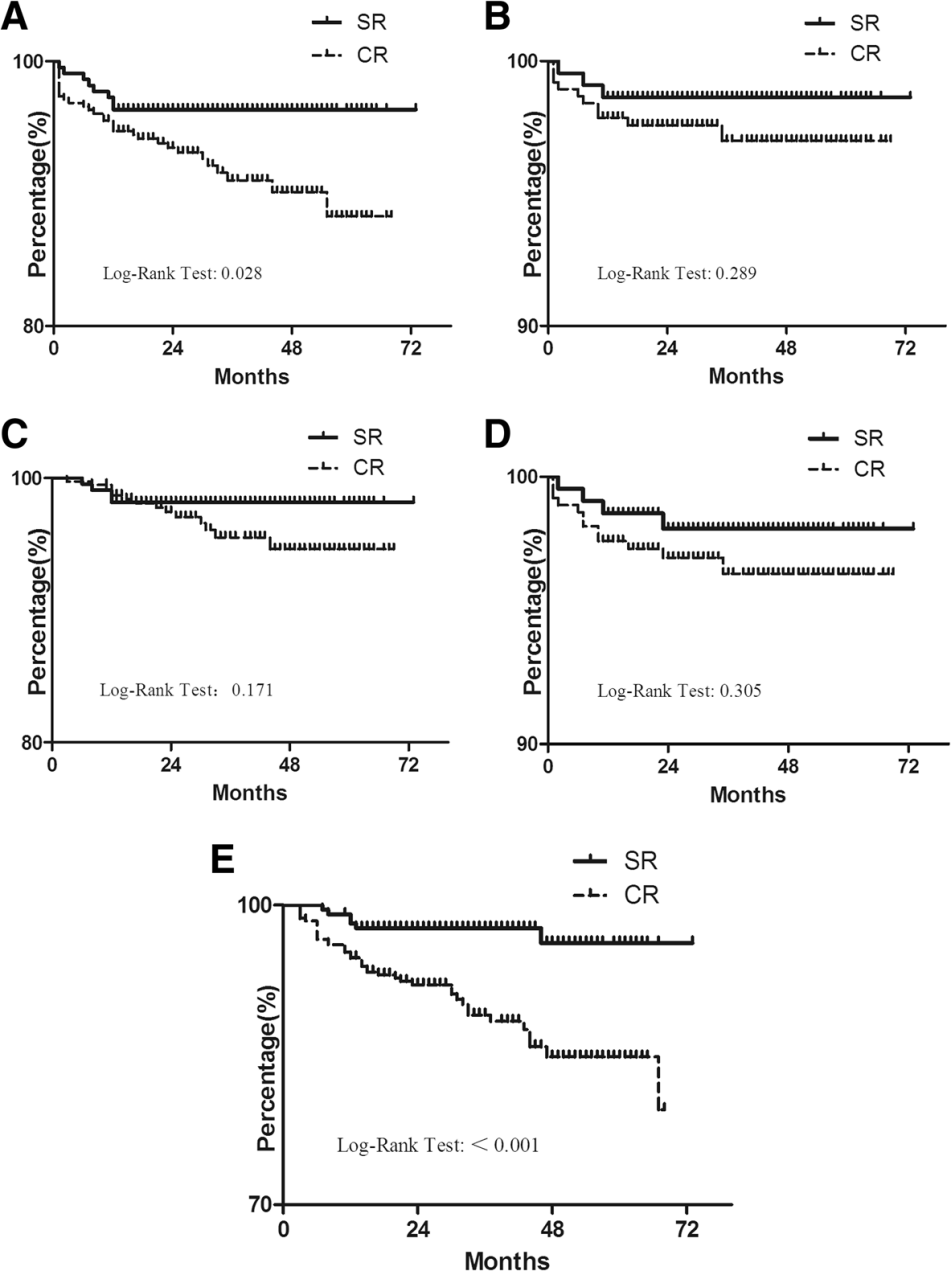
Kaplan-Meier survival curves free from (**a**) cardiac mortality/nonfatal reinfarction, (**b**) cardiac mortality, (**c**) nonfatal reinfarction, (**d**) all-cause mortality, (**e**) unplanned repeat revascularization according to the different groups. SR, staged complete revascularization group; CR, culprit-only revascularization group

All patients were followed for a mean duration of 35 months (12–71 months). The length of follow-up in the CR group was 34 months (12–69 months), and was 36 months (12–71 months) in the SR group. During the follow-up period, 31 events of cardiac mortality/nonfatal myocardial reinfarction events, 17 events of cardiac mortality, 14 events of nonfatal myocardial reinfarction, 19 events of all-cause mortality, and 42 events of unplanned repeat revascularization were observed in the CR group; 8 events of cardiac mortality/nonfatal myocardial reinfarction, 4 events of cardiac mortality, 4 events of nonfatal myocardial reinfarction, 5 events of all-cause mortality, and 9 events of unplanned repeat revascularization were observed in the SR group. The composite of cardiac mortality or nonfatal reinfarction was significantly lower in the SR group compared with the CR group [HR: 0.427, 95 % CI: 0.196–0.929, *P* = 0.032], and unplanned repeat revascularization showed a similar trend [HR: 0.349, 95 % CI: 0.170–0.717, *P* = 0.004] (Fig. 2; Table 3). After adjusting for covariates (Model 1), the SR group was still associated with a lower rate of the composite of cardiac mortality or nonfatal reinfarction [HR: 0.430, 95 % CI: 0.197–0.940, *P* = 0.034] and unplanned repeat revascularization [HR: 0.343, 95 % CI: 0.166–0.708, *P* = 0.004] compared with the CR group (Table 3). There were no statistically significant differences in the other endpoints between the two groups (Table 3). Periprocedure-related complications were not significantly different (Table 4).

**Table 3:** Univariate and multivariate analysis of the effects of different treatment strategies at follow-Up, *N* (%)

	No. patients with event		Univariate analysis		Multivariate analysis*	
	CR	SR	HR (95 % CI)	*P*	HR (95 % CI)	*P*
Primary end points						
Cardiac mortality/Nonfatal reinfarction	31 (8.1)	8 (3.6)	0.427 (0.196–0.929)	0.032	0.430 (0.197–0.940)	0.034
Secondary end points						
Cardiac mortality	17 (4.5)	4 (1.8)	0.400 (0.135–1.190)	0.100	0.440 (0.147–1.319)	0.143
Nonfatal reinfarction	14 (3.7)	4 (1.8)	0.467 (0.153–1.418)	0.179	0.442 (0.143–1.365)	0.156
All-cause mortality	19 (5.0)	5 (2.3)	0.442 (0.165–1.185)	0.105	0.489 (0.181–1.321)	0.158
Unplanned repeat revascularization	42 (11.0)	9 (4.1)	0.349 (0.170–0.717)	0.004	0.343 (0.166–0.708)	0.004

*Adjusted for age, diabetes, hypertension, Killip class II/III on admission, systolic blood pressure on admission, heart rate on admission, symptom to balloon time, and anterior MI

**Table 4:** Periprocedure-related complications, *N* (%)

	CR (*n* = 382)	SR (*n* = 220)	*P*
BARC 3 or 5 bleeding	2 (0.5)	4 (1.8)	0.124
Contrast-induced nephropathy	13 (3.4)	5 (2.3)	0.433
Stroke	3 (0.8)	0	0.188
Acute or subacute stent thrombosis	1 (0.3)	0	0.448

## Discussion

The present study determined the effects of different treatment strategies on STEMI patients with MVD in a real-world clinical setting. The main findings were as follows: (1) staged complete revascularization significantly reduced not only the rate of the composite of cardiac mortality or nonfatal reinfarction, but also the need for unplanned repeat revascularization; (2) no significant differences in all-cause mortality, cardiac mortality or nonfatal reinfarction were observed between the treatment strategies; (3) staged complete revascularization did not significantly increase periprocedure-related complications.

Toyota et al. analyzed 1311 STEMI patients with MVD undergoing P-PCI from the CREDO-Kyoto AMI Registry in Japan (681 in the staged PCI group versus 630 in the culprit-only PCI group), and reported that staged PCI was associated with a lower 5-year composite of cardiac mortality and myocardial infarction compared with culprit-only PCI [HR: 0.67, 95 % CI: 0.44–0.99, *P* = 0.045] [19]. Our findings also showed a lower composite of cardiac mortality and nonfatal reinfarction in the SR group. A similar conclusion was found in the CvLPRIT and DANAMI-3—PRIMULTI trials [10, 11]. However, no studies have found significant differences in cardiac mortality [8–12, 19] between the treatment groups. Furthermore, most studies [8–12, 17, 19, 20] found no significant differences in nonfatal reinfarction, except for the PRAMI trial [9] and a recent meta-analysis [23]. Our study also failed to find significant differences in cardiac mortality and nonfatal reinfarction between the two groups. It was demonstrated that staged complete revascularization significantly reduced the need for unplanned repeat revascularization; however, the Japanese study [19] and CvLPRIT trial [10] found no significant differences, and the proportion of patients with three-vessel disease may have played an important role. There was a higher proportion of three-vessel disease in the CR group in our study than in the other two previous studies. In other words, the higher the proportion of three-vessel disease, the higher the proportion of ischemia-driven unplanned repeat revascularizations. Meta-analyses have also confirmed that multivessel PCI will reduce the need for repeat revascularization [22–24]. Different to other studies [17–19], our study found no significant differences in all-cause mortality. It is possible that the follow-up duration in our study was too short to detect significant differences in all-cause mortality: 3-year follow-up in our study, compared with 5-year and 7-year follow-up in the other two studies [18, 19]. In addition, the sample size in our study was relatively small, 602 individuals compared with 8822 and 1311 in the other two studies [18, 19]. Accordingly, adequately powered randomized studies should be performed to obtain meaningful conclusions, such as in the COMPLETE trial (ClinicalTrials.gov NCT01740479).

The safety concerns regarding complete revascularization include the risk of procedural complications, longer procedural time, contrast nephropathy, and stent thrombosis which may increase in a prothrombotic and proinflammatory state in the presence of STEMI. Despite this, our study showed no increase in major bleeding, contrast-induced nephropathy, stroke, acute or subacute stent thrombosis. This was consistent with previous studies [8, 10–12, 19].

There are still several problems related to the treatment of STEMI. First, is staged complete revascularization better than "one-time" complete revascularization? While analysis from the HORIZONS-AMI trial preferred staged complete revascularization [15], other studies found "one-time" complete revascularization safe and effective [20, 21]. Second, what is the appropriate timing of staged revascularization? Different studies had different time cut-off points; however, no study could confirm a favored time cut-off point. Third, should fractional flow reserve (FFR) or a non-invasive physiological stress test be used to determine indications for staged revascularization in addition to angiography? FFR measurements of non-culprit lesions could be performed immediately [28] or several days or weeks [7] after treatment of the culprit vessel. To date, studies with FFR as the reference [11, 13, 14] did not have clearer conclusions than those without FFR as the reference [8–10]. The COMPARE ACUTE trial (ClinicalTrials.gov NCT01399736), an ongoing prospective randomized study comparing a FFR-guided multivessel PCI undertaken during primary PCI of the culprit vessel only, may help us to define the role of FFR in STEMI patients with MVD. Fourth, do the benefits extend to non-culprit stenoses of less than 70 % or 50 %? The level of non-culprit stenosis at which the risks of PCI surpass the benefits is still uncertain. In addition to FFR, intracoronary imaging such as an intravascular ultrasound study (IVUS) and optical coherence tomography (OCT) could be useful tools for non-culprit lesion revascularization. IVUS and OCT could help us describe *in vivo* the pathological morphology of plaque associated with an impaired myocardial blush and slow flow leading to a worse prognosis [29]. As for the use of IVUS and OCT, a per-patient tailored therapy may be achieved.

### Limitations

This study had several limitations. First, the study was retrospective and observational, thus potential confounders and selection bias could not be completely adjusted. Second, this was a single center study. Third, the significance of non-culprit lesions was assessed only on angiography, and ischemia tests such as FFR were absent. Fourth, the long symptom to balloon time in this study may have had an impact on the study results, as analysis of the HORIZONS-AMI trial results suggested that a delay in mechanical reperfusion therapy during STEMI is associated with greater injury to the microcirculation [30], and another study showed that a symptom-onset-to-balloon time >4 h was an independent predictor of one-year mortality [31]. Finally, the incidence of the primary composite end-point was quite low during the follow-up period. The low number of events may be a limitation in the overall interpretation of the study results.

## Conclusions

In STEMI patients with MVD, staged complete revascularization for angiographically significant non-culprit lesions was associated with a significantly lower composite of cardiac mortality or nonfatal reinfarction and unplanned repeat revascularization.

## Abbreviations

bpm: Beats per minute
CI: Confidence interval
CR: Culprit-only revascularization group
h: Hour
HR: Hazard ratio
LVEF: Left ventricular ejection fraction
MI: Myocardial infarction
MVD: Multivessel disease
PACS: Picture Archiving and Communication Systems
P-PCI: Primary percutaneous coronary intervention
RCT: Randomized controlled trial
SR: Staged complete revascularization group
STEMI: ST-segment elevation myocardial infarction
TIMI: Thrombolysis In Myocardial Infarction
